# Supplementary material for: Molecular Conformer Search with Low-Energy Latent Space
Source: J Chem Theory Comput. 2022 Jun 13;18(7):4574–85. doi: 10.1021/acs.jctc.2c00290 (PMC9281398; doi:10.1021/acs.jctc.2c00290)
Supplement: Supplementary file 1 — ct2c00290_si_001.pdf [file ct2c00290_si_001.pdf]

Molecular Conformer search with low-energy latent space (SI)

Xiaomi Guo,[†] Lincan Fang,[‡] Yong Xu,[†] Wenhui Duan,[†] Patrick Rinke,[‡] Milica Todorović,[*,¶] and Xi Chen[*,‡]

†*Department of Physics, Tsinghua University, Beijing, China*

‡*Department of Applied Physics, Aalto University, Espoo, Finland*

¶*Department of Mechanical and Materials Engineering, University of Turku, FI-20014 Turku, Finland*

E-mail: milica.todorovic@utu.fi; xi.6.chen@aalto.fi

Analysis of CYS800 data set (CYS800)

Figure S1 shows the distributions of dihedral angles and scaled energy of the CYS800 data set. The size of the data set is 800. The histogram shows that d_1 and d_3 are uniformly distributed in [0°, 360°], but the distributions of d_2, d_4, and d_5 have three, two and two peaks. Only 5 data in the test set has the scaled energy above 1.0. And most data has energy around the peak at -0.25.

The progression of training loss

Figure S2 shows the training loss of the CYS800 data set changes with training epochs. The VAE models are trained by using $\lambda = 0.01, \beta = 0$ and $layersize = 128$. The blue, red and green lines represent the total loss, the reconstruction loss, and the KL divergence. The KL

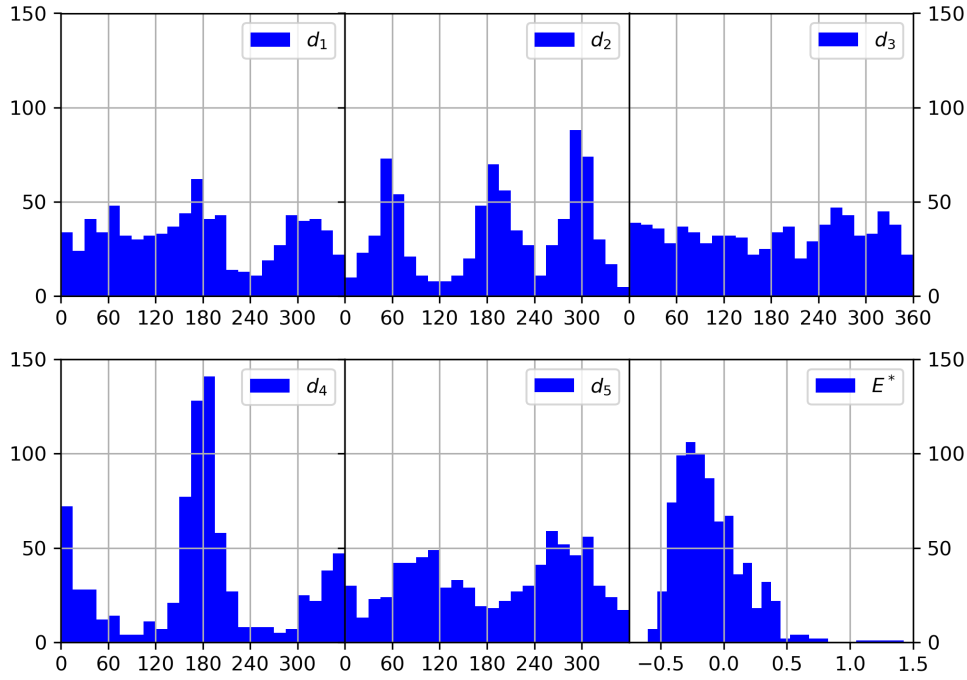

Figure S1: The dihedral angles and energy distribution of test set.

divergence keeps stable after 10000 epochs. The reconstruction loss decreases to 0.03 at the first 50000 epochs and stabilizes after 100000 epochs. We select 100000 epochs to ensure the convergence of the network training.

The latent-space data distributions with different λ

Figure S3 shows the latent-space data distributions of the CYS800 data set with different λ values. We plot the latent-space data (μ_{i1}, μ_{i2}) and the color represents the scaled energy of the corresponding molecular structure. For $\lambda = 0$, the VAE decays to a normal auto-encoder and the data distribution is non-uniform: data points in the center of latent space are closed to each other, while other data points are sparsely distribute in the rest of latent space. When λ increases a small amount to 0.001, the scale of latent space drops (see axis labels in Figure S3). When $\lambda = 0.01$, the data distribute even more uniformly inside a circle. When λ increases to 0.1 the latent space restructures into parallel lines. Further increasing

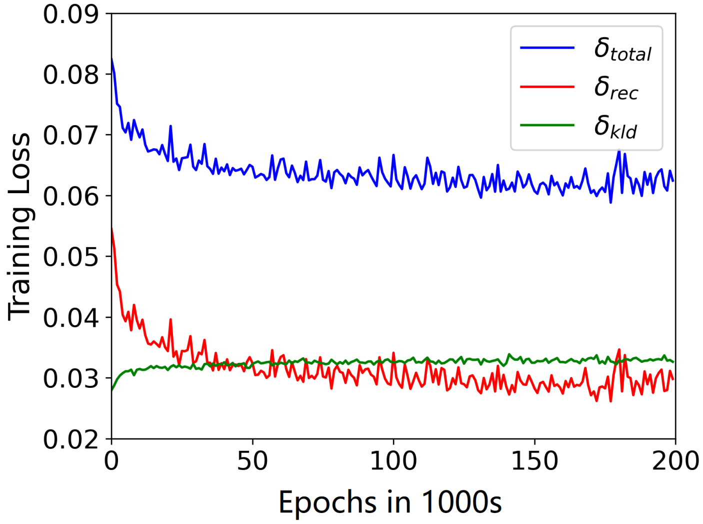

Figure S2: The progression of training loss with training epochs.

λ compresses latent space and it finally collapses into a single point for $\lambda \sim 1$.

Statistic analysis of cysteine results

We extract the local minima from the GP at different iterations and use them to initialize DFT geometry optimization. The candidate structures are further optimized with DFT structure relaxation and the results are shown in Figure S4. Some targets found at certain iteration might be missed in following iterations. For example, the second target (sorted by energy) is found at iteration 15 and 20 but missed at iteration 25. This suggests us not to keep only the result of the final energy model but the accumulative results.

Figure S5 shows how the training loss, the latent-space scale and the energies of samples changes during the data generation step for cysteine. For the same β, the KL Divergence remains stable among iterations because it is controlled by the hyperparameter λ. The fixed λ also limits the value of latent-space scale L in the range $1.40 - 1.55$, which is closed to the latent-space scale $L \sim 1.47$ of the test set. The reconstruction loss and the total loss increase as the iteration increases, but the increasing rate and the start value are not related to the values of β.

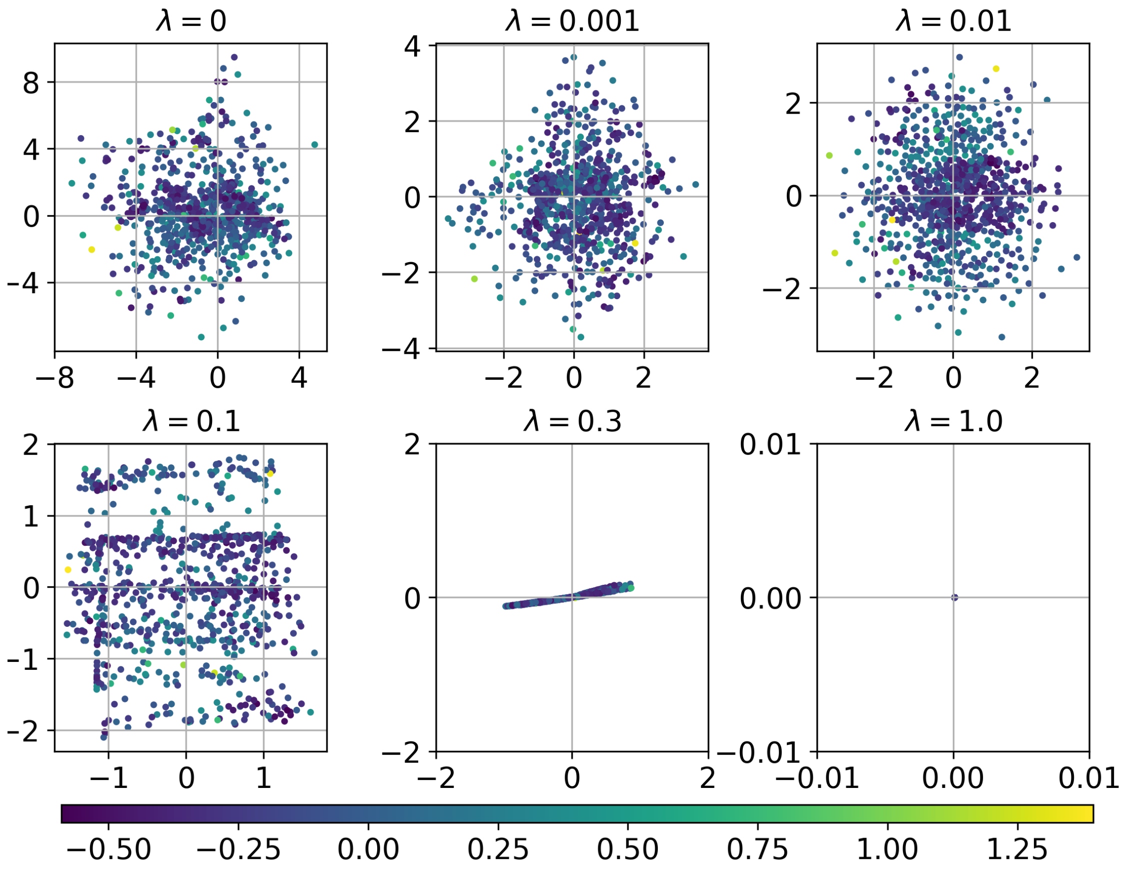

Figure S3: The latent-space data distributions of the CYS800 data set with different λ.

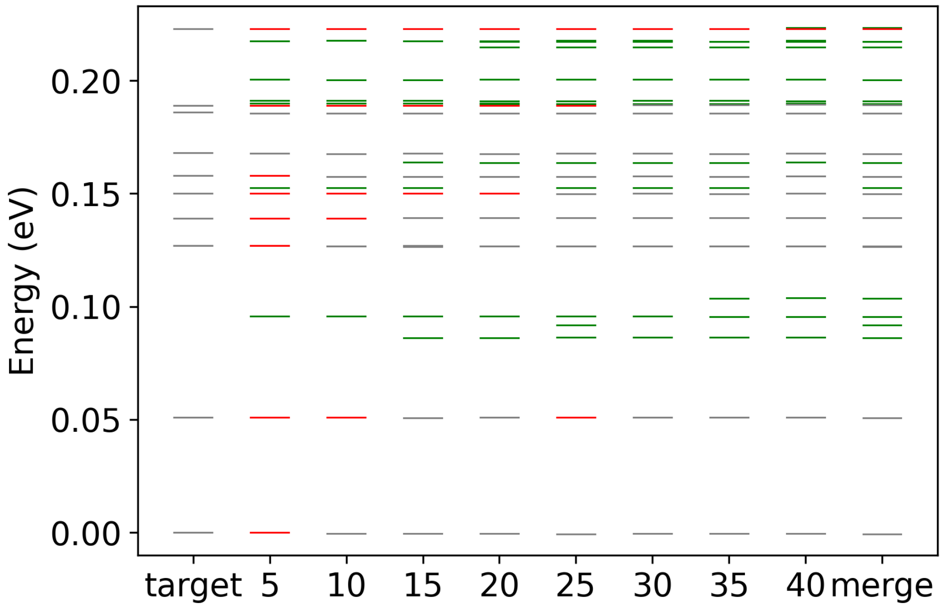

Figure S4: The targets found at different iterations.

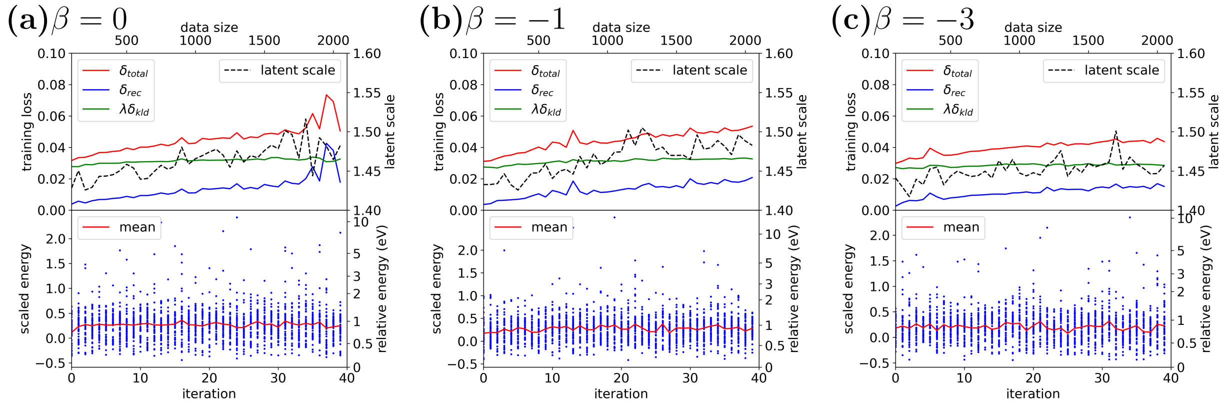

Figure S5: The training loss, latent-space scale, and energies of samples during the data generation. (a)$\beta = 0$ (b)$\beta = -1$ (c) $\beta = -3$.

Statistic analysis of GFA results

Figure S6 shows the reconstruction loss, the latent-space scale and the energies of samples during the data generation step for GFA . For each run, both the reconstruction loss and the latent-space scale slowly increase as the iteration steps increase, in which the workflow with $\beta = -3$ has a minimal increase rate. The KL-Divergence has a stable value among iterations, but the value for $\beta = -3$ is only half of which for $\beta = 0$ or -1. The latent-space scale L is about 1.25 for $\beta = -3$, which is smaller than $1.47 - 1.61$ for $\beta = 0$ and $1.45 - 1.52$ for $\beta = -1$. The average energy of samples has $\sim 10\%$ fluctuation among iterations but decreases about 1 eV when β changes from 0 to -3. More low-energy samples are acquired for $\beta = -3$ than $\beta = -1$ or 0.

Similar GFA conformers

Figure S7 shows three very similar GFA conformers. The blue rectangles show the difference between GFA 06 and GFA 11 is at the $-C_6H_6$ branch (benzene ring), which causes the energy difference 1.7 meV. The orange rectangles show the difference between GFA 11 and GFA 08 is at the $-CH_2NH_2$ branch, which causes the energy difference 1.9 meV.

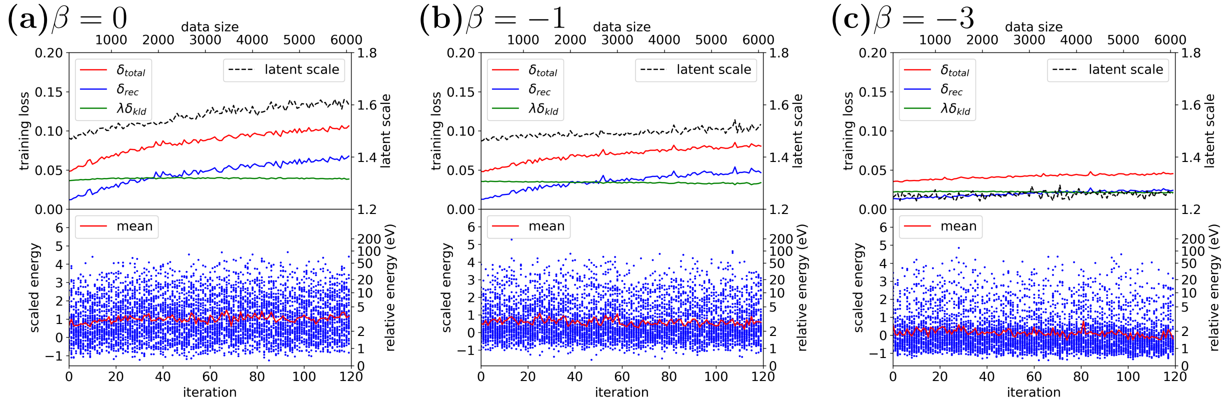

Figure S6: The training loss, the latent-space scale, and the energies of samples during the data generation. (a)$\beta = 0$ (b)$\beta = -1$ (c) $\beta = -3$.

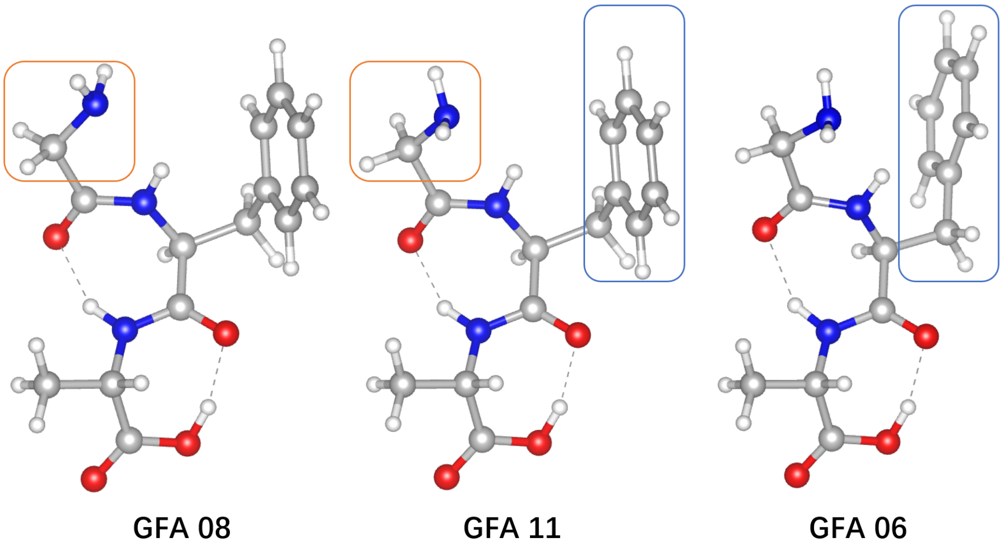

Figure S7: Three very similar GFA conformers: GFA 08, GFA 11 and GFA 06. The orange rectangles show the main difference between GFA 08 and GFA 11. The blue rectangles show the main difference between GFA 11 and GFA 06.

Detailed results of WG, GGF and WGG

Figure S8 shows the accumulative results for WG (7d), GGF (9d) and WGG (9d).

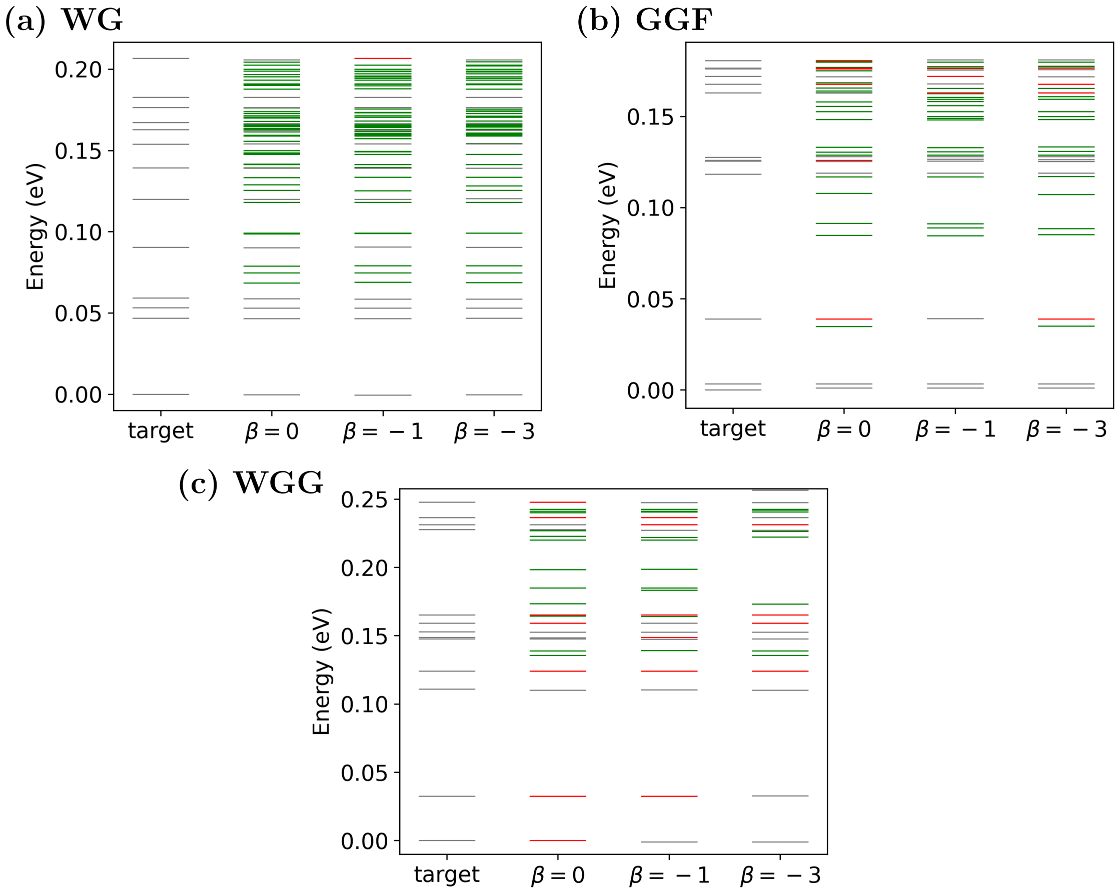

Figure S8: The accumulative results for (a) WG (7d), (b) GGF (9d) and (c) WGG (9d).

Detailed results of real space search

Algorithm S1 shows the real space search workflow.

Figure S9 is the comparison of the GFA results from real space search workflow (Algorithm 2) and the LOLS. The real space search workflow acquired 6000 and 10000 samples. LOLS acquired 6000 samples with different values of β. The real space search workflow missed the top eight lowest energy targets with 6000 samples and remained to perform poorly up to 10000 samples.

Algorithm S1 Real space search workflow

```
Require: noise, M, k
DataPool = ∅
StableComformers = ∅
for i = 1 ... M do
    vector = RamdomVector()
    atoms = Vec2Atoms(vector)
    energy = DFTEnergy(atoms)
    DataPool ← {vector, energy}
    if (i ≡ 0  mod k) then
        Initialize(GP, DataPool)
        Optimize(GP, noise)
        for vector, energy ∈ DataPool do
            Optimize(vector, GP)
            atoms = Vec2Atoms(vector)
            Optimize(atoms)
            StableComformers ← atoms
        end for
    end if
end for
Return StableComformers
```

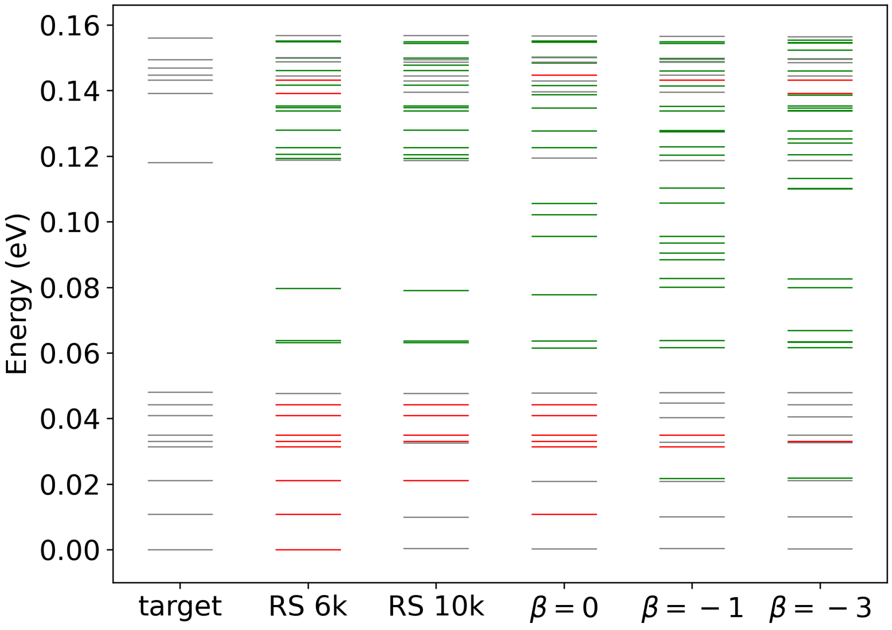

Figure S9: Comparison of LOLS and real space search workflow on GFA. RS 6k and RS 10k represent the results of the real space search workflow with 6000 and 10000 samples.

Analysis of the final data pools of cysteine, WG, and GFA

Figure S10 gives the relationship between the reconstruction error (MAE of dihedral angles) and the scaled energy in the last iteration of LOLS for cysteine. A negative β makes the data with lower energy have less reconstruction loss and causes the workflow to acquire more lower-energy data.

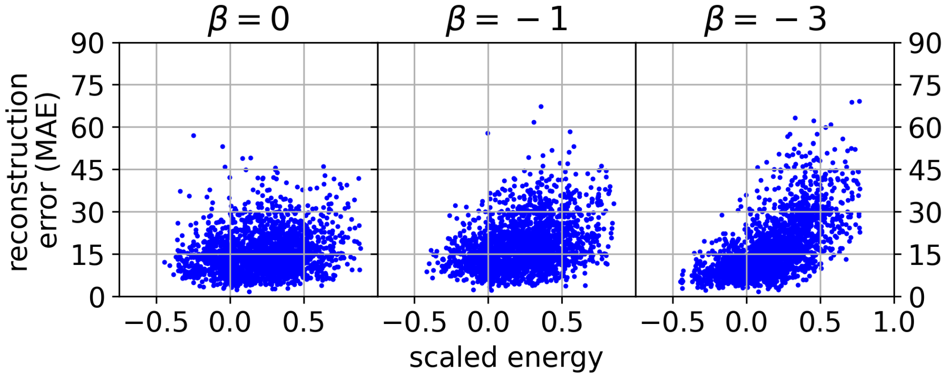

Figure S10: The relationship between the reconstruction error (MAE of dihedral angles) and the scaled energy in the last iteration of LOLS for cysteine.

Figure S11 shows the energy distribution of the data in the last LOLS iteration for cysteine, WG and GFA. The iteration number for Cysteine, WG, and GFA are 40, 120, and 120. The energy zero point is set to the global minima of the molecules. $\beta = -3$ makes LOLS obtain more lower energy samples. And the tendency becomes more significant as the dimension increases from five to nine. On the other hand, the ratio of higher energy (> 4 eV) increases as the dimension increases. The reason is that molecules with more atoms have a higher probability of collisions between atoms which causes high DFT energies

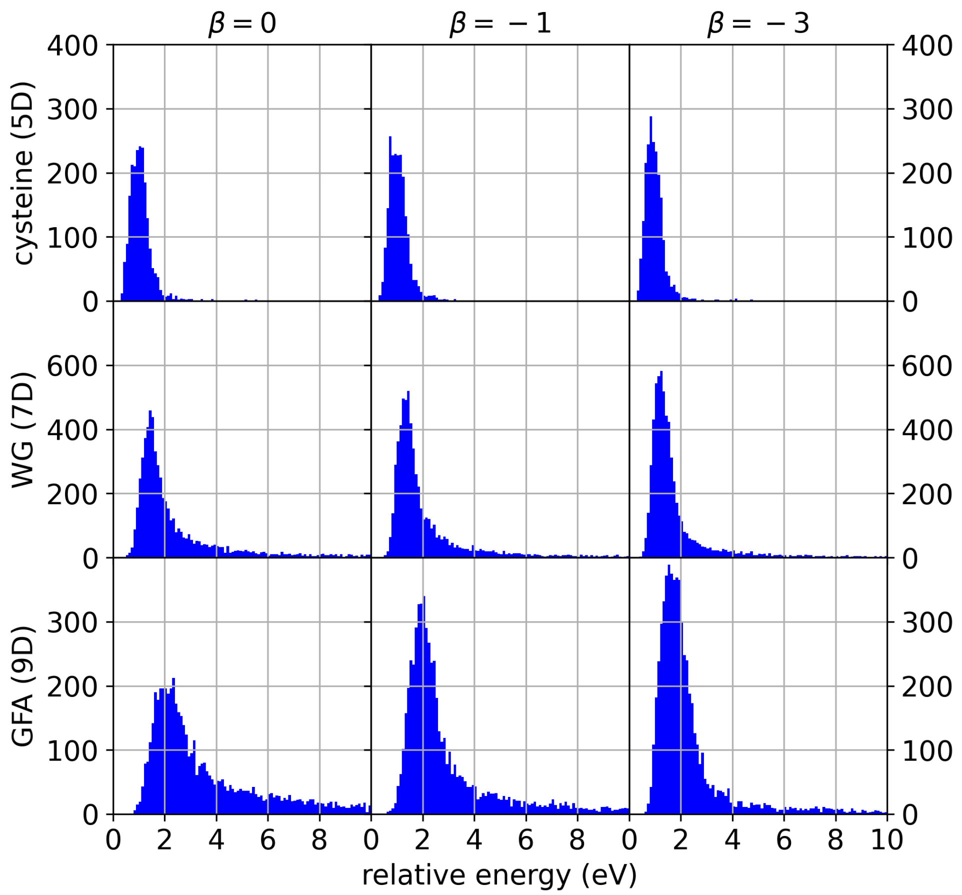

Figure S11: The energy distribution of the data in the last LOLS iteration for cysteine, WG and GFA.
